# Supplementary material for: Reasons for (not) choosing dental treatments—A qualitative study based on patients’ perspective
Source: PLoS One. 2022 May 25;17(5):e0267656. doi: 10.1371/journal.pone.0267656 (PMC9132305; doi:10.1371/journal.pone.0267656)
Supplement: S2 File — (PDF) [file pone.0267656.s002.pdf]

HECOR Gesundheitsökonomisches Zentrum Berlin

Technische Universität Berlin

Participant information

Dear participant,

Thank you for participating in the discussion group "Patients' preferences for choosing dental services"!

In the following we would like to inform you about the discussion group:

1. Participation in this discussion group is voluntary. Your participation can be withdrawn at any time without giving reasons and without any disadvantages for you. You can leave the current discussion group at any time. This has no influence on the payment of your compensation.

2. Content: The aim of this discussion group is to identify reasons which influence you as a potential patient in your choice for or against a dental treatment and, if applicable, have influenced you in the past. It is also intended to highlight what, if any, consequences your choice may have had, such as unplanned follow-up treatments or an installment to pay the treatment costs.

3. Aim: The results of several discussion groups are to be analyzed and published anonymously. The results will be used to develop a questionnaire that captures preferences of (potential) patients and their willingness-to-pay for different dental treatment alternatives in Germany. The implementation and evaluation of the discussion groups are part of a research project funded by the German Federal Ministry of Education and Research. The aim is to use the identified patients' preferences to make suggestions for improvements and recommendations for action to decision-makers in health policy to enhance dental care from patients' perspective.

4. Each discussion group is limited to three hours, recorded on tape and anonymized in a subsequent transcription. Your statements will be used by determining categories of patient preferences and counting them, and quoting excerpts in publications if applicable (e.g., "I chose a dental crown because [...]."(FG3 TN2).)". Here FG stands for "focus group" and TN for "participant". Thus, different participants can be quoted anonymously in excerpts. In addition, personal data will be collected from you by means of a questionnaire, that will be published anonymously and exclusively in aggregated form. It is not possible to draw conclusions about you as a participant. The tape recordings and the personal data will be kept separately and deleted after completion of the study and a retention period of ten years. You can exercise your right of withdrawal within 14 days from the day after the discussion group and request deletion of your raw data. To do so, please contact Ms. Susanne Felgner. She can be reached at 030/31428701 or by e-mail at susanne.felgner@tu-berlin.de.

5. In order to maintain the anonymity of any data, all participants will be addressed exclusively by their first name or an alternative (self-chosen) name during the discussion group. All participants of the discussion group, including the moderator, co-moderator and student assistant, are bound to secrecy. You are allowed to share experiences at home, but not by mentioning a name. Any violation is punishable by law. Of course, no personalized data will be communicated to institutions such as health insurance companies or physicians, etc. Participation in the discussion group will not result in any individual advantages or disadvantages for you in terms of (dental) medical care. This enables you to openly address points relevant to you on the topic discussed.
