# Supplementary material for: Reasons for (not) choosing dental treatments—A qualitative study based on patients’ perspective
Source: PLoS One. 2022 May 25;17(5):e0267656. doi: 10.1371/journal.pone.0267656 (PMC9132305; doi:10.1371/journal.pone.0267656)
Supplement: S3 File — (PDF) [file pone.0267656.s003.pdf]

Discussion group *dd/mm/yyyy*

Dear participant* ________________________________,

we ask you to answer the following questions. Your answers will be treated absolutely confidentially.

(1) What type of health insurance are you a member of?

(1.1) Statutory health insurance (SHI) ☐ yes ☐ no ☐ I don't know

(1.1.1) I have compulsory insurance ☐ yes ☐ no ☐ I don't know

(1.1.2) I am voluntarily insured ☐ yes ☐ no ☐ I don't know

(1.1.2) I have family insurance ☐ yes ☐ no ☐ I don't know

(1.2) Private health insurance (PHI) ☐ yes ☐ no ☐ I don't know

(2) Do you have a dental supplementary insurance? ☐ yes ☐ no ☐ I don't know

(3) Do you receive allowances in case of need, e.g. for civil servants and soldiers? ☐ yes ☐ no ☐ I don't know

(4) Do you own a "Bonus booklet"? ☐ yes ☐ no ☐ I don't know

(4.1) Have you ever benefited from your "Bonus booklet", i.e., has it ever reduced the amount of your out-of-pocket payment? ☐ yes ☐ no ☐ I don't know

(5) Do you use a "Bonus program" of your health insurance? ☐ yes ☐ no ☐ I don't know

(5.1) Have you ever benefited from the "Bonus program", i.e., has it ever reduced the amount of your out-of-pocket payment? ☐ yes ☐ no ☐ I don't know

(6) Have you ever undergone a dental treatment for that you had to make an out-of-pocket payment? ☐ yes ☐ no ☐ I don't know

(7) Has your dentist ever presented you different alternatives in advance of a dental treatment (and related costs), e.g., tooth-filling with high-quality plastic instead of amalgam? ☐ yes ☐ no ☐ I don't know

* Your first name or self-chosen name by which you were addressed in the discussion group

(8) How old are you? _____years

(9) What is your gender? ☐ female ☐ male ☐ other

(10) What is your highest educational qualification?

- (Technical) university degree ☐
- Vocational training ☐
- Graduation after 13 school years (A-level) ☐
- Graduation after 10 school years ☐
- Graduation after 9 school years ☐
- No school graduation ☐
- No answer ☐
- Other: ______________________________________ ☐

(11) Are you currently employed?

- Yes, I am fulltime employed ☐
- Yes, I am part-time employed ☐
- I am a student ☐
- No, I am unemployed ☐
- No, I receive pension due to illness ☐
- No, I receive pension due to age ☐
- Other: ______________________________________ ☐

(12) What is your net household income per month?

- < 500€ ☐
- 500 to < 750€ ☐
- 750 to < 1.000€ ☐
- 1.000 to < 1.500€ ☐
- 1.500 to < 2.500€ ☐
- 2.500 to < 3.500€ ☐
- 3.500 to < 4.500€ ☐
- 4.500 to < 5.500€ ☐
- 5.500 to < 6.500€ ☐
- > 6.500€ ☐

(7) How did you hear about the discussion groups?

- Flyer / notice at TU Berlin ☐ in grocery store ☐
- Newspaper advert in “Berliner Woche” ☐
- Advert at “eBay Kleinanzeigen” ☐
- Post at “Facebook” ☐
- E-Mail from TU Berlin mailing list ☐
- Other: ______________________________________ ☐

We are interested in your opinion:

What did you particularly like about the discussion group? ________________________________

____

____

____

What did you not like about the discussion group at all? __________________________________

____

____

____

Thank you for participating!
