# Supplementary material for: Reasons for (not) choosing dental treatments—A qualitative study based on patients’ perspective
Source: PLoS One. 2022 May 25;17(5):e0267656. doi: 10.1371/journal.pone.0267656 (PMC9132305; doi:10.1371/journal.pone.0267656)
Supplement: S4 File — (PDF) [file pone.0267656.s004.pdf]

HECOR Gesundheitsökonomisches Zentrum Berlin

Technische Universität Berlin

Informed consent

Discussion group: "Patients' preferences for choosing dental services".
Research project: "Medical Devices: Incentives and Impact of Regulatory Instruments (MeDIoRI)".

I have been informed about content and purpose of the discussion group. I have read and understand the information provided. I have received a copy of this consent form.

My participation in the discussion group is voluntary. My attention has been drawn to the fact that participation can be withdrawn at any time without giving reasons. I can leave the ongoing discussion group at any time without incurring any disadvantages.

I further agree that the discussion group will be recorded on tape and that my personal data will be kept anonymously for ten years until it is deleted. I can exercise my right of withdrawal within 14 days from the day after the discussion group and request deletion of my raw data. To do so, I contact the contact address provided (see bottom of page). In addition, I agree that the data collected may be used, subject to anonymity, in the context of the above-mentioned research project and related publications and lectures. In the case of lectures, no tape recordings will be used but, if necessary, only anonymized quotes based on the anonymized transcripts. Personal data will only be presented in aggregated form. It is not possible to draw conclusions about individual persons. In order to preserve the anonymity of any data, all participants will be addressed exclusively by their first name or an alternative (self-chosen) name during the discussion group. I must maintain confidentiality about the discussion group. I may share experiences from the discussion group at home, but not in a personalized form. Any violation is punishable by law. Of course, no personalized data will be communicated to health insurance companies, physicians, etc.

Date, surname and first name of participant	Signature of participant
______________________________	______________________
Date, surname and first name of moderator	Signature of moderator
______________________________	______________________
Date, surname and first name of co-moderator	Signature of co-moderator
______________________________	______________________
Date, surname and first name of student assistant	Signature of student assistant
______________________________	______________________

Contact address:

Susanne Felgner, H80, Straße des 17. Juni 135 in 10623 Berlin,
Tel.: 030/31428701, e-mail: susanne.felgner@tu-berlin.de
