# Supplementary figures and images for: Reasons for (not) choosing dental treatments—A qualitative study based on patients’ perspective
Source: PLoS One. 2022 May 25;17(5):e0267656. doi: 10.1371/journal.pone.0267656 (PMC9132305; doi:10.1371/journal.pone.0267656)

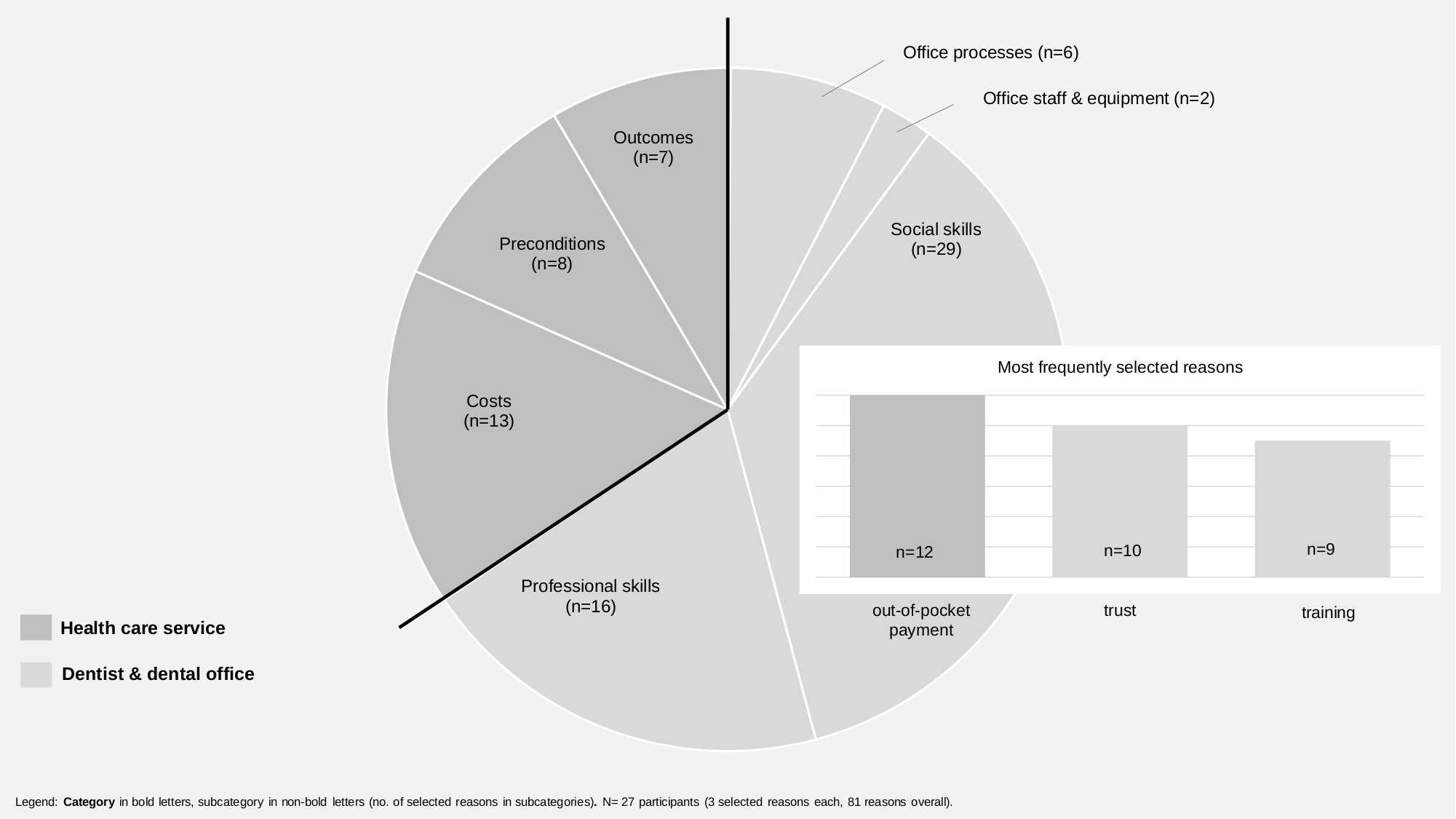

Supplement: S6 File — (TIFF) [file pone.0267656.s006.tiff]

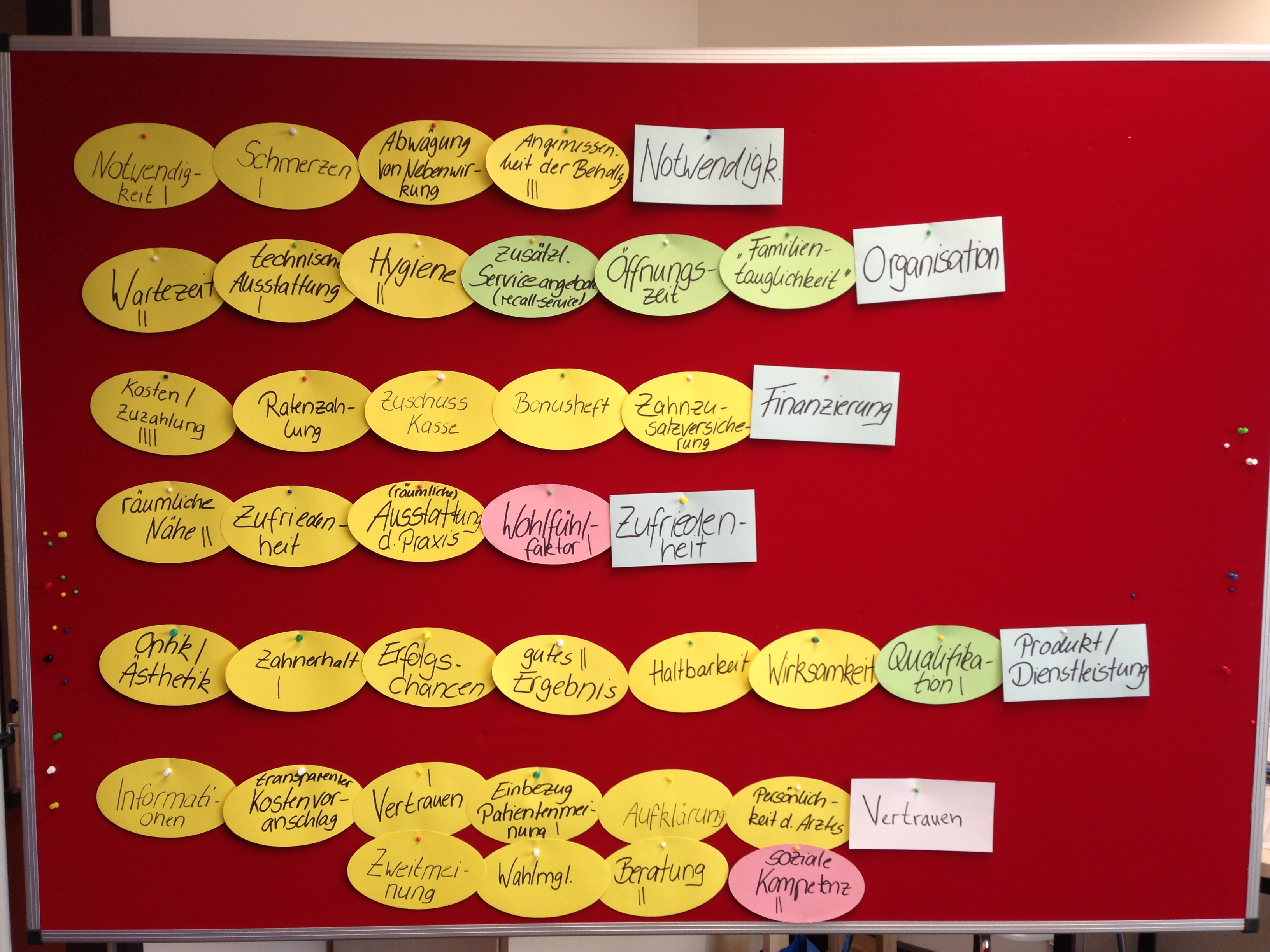

Supplement: S7 File — (TIFF) [file pone.0267656.s007.tiff]
