# Supplementary material for: Reasons for (not) choosing dental treatments—A qualitative study based on patients’ perspective
Source: PLoS One. 2022 May 25;17(5):e0267656. doi: 10.1371/journal.pone.0267656 (PMC9132305; doi:10.1371/journal.pone.0267656)
Supplement: S1 Table — (DOCX) [file pone.0267656.s012.docx]

**S1 Table. Interview guide used in focus groups.**

| **Structure** | **Content, including questions** | **Moderator notes** |
| --- | --- | --- |
| **I Reception** | - Reception of the participants (offering coffee, etc.) - Assigning their seat and issuing participants’ information document, informed consent | - |
| **II Welcome/Presentation of the department and the research project** | - Department of Health Care Management at the Technische Universität Berlin - Research project: We investigate   - effects of **regulatory measures** on the **physicians’ utilization of (innovative) procedures and medical devices**, and   - **factors** influencing physicians and patients in their **choice for or against** a medical procedure and device. - For that reason, we have invited you as our experts to this discussion group. What we would like to know from you:   - Which factors influence you as a patient when choosing or NOT choosing a dental treatment;   - i.e.: Why do you **decide for** a dental treatment and why do you **decide against** a dental treatment? - Introduction of moderator, co-moderator, and student assistant | - |
| **III Explanation of interview agenda** | - Interview consists of two parts, and a 20-minute break between   1. Part:   - - Self-introduction of each participant   - Report on experiences at the dentist regarding:     - Reasons for choosing a dental treatment and     - reasons for NOT choosing a dental treatment.   Break:   - - Snacks and drinks offered in the hallway for the participants   - Moderator, co-moderator, and assistant:     - Summarized given statements,     - transform statements to reasons and     - form categories for reasons.   2. Part:   - - Presentation of identified reasons to the participants, formulated as expectation.   - Do participants agree on identified reasons? / What is missing?   - Ranking by each participant: Three “most important” reasons of choice for a dental treatment. - Closing:   - Handing out questionnaires, signing informed consent, and payment of 20€ compensation | How to bring back participants who digress:  “[name], may I interrupt you and ask you to briefly list your arguments again?”  “[name], may I briefly summarize ..., did I understand that correctly?”  “[name], may I ask you to return to the initial question?” |
| **IV Formalities and additional instructions** | - Participants’ information document and informed consent:   - Participation in the interview is voluntary,   - you can leave the interview at any time without giving reasons,   - the interview is limited to 3 hours and audio-taped,   - everything you say and any data collected from you will be analyzed anonymously. No conclusions can be drawn about your person.   - To ensure anonymity all participants will be called by their first name,   - or if you do not want to be called by your name, please choose a name.   - All participants of the discussion group are bound to secrecy. We must point out that any breach of the rules is punishable by law. - Declaration of consent:   - The points mentioned are also listed in the informed consent and you agree to them with your signature. - Even if we address each other by our first names or an alternatively chosen name, we would like to remain formal and use the form of address “Ms./Mrs.” or “Mr.”. - In addition, we would like to ask you to keep the noise level low (e.g., when leaving the room) to not impair the quality of the audio-recording. - We appreciate if you actively participate in the interview! - What and how much you disclose about yourself is up to you, of course. - You can have snacks and drinks and leave the room at any time. - The way to the bathrooms is signposted. - After the interview, please stay for a while to fill out the participant questionnaire and receive your compensation. | - |
| **V Introduction round/ Opening questions (Opening & Introductory question)** | - Please introduce yourself briefly with your first name, or a chosen name and mention your age. Please write your name on the prepared name tag! - What are your experiences at the dentist?   Have you ever decided for or against a dental treatment?   - Have you ever been offered a dental treatment with a co-payment?  [e.g., tooth-filling made of so-called high-quality plastic material, or a dental crown made of ceramics] | Example for context:  Do you go to the dentist often?  Have you ever had to undergo a dental treatment that goes beyond the annual check-up? |
| **VI Transition questions: identification of reasons (Transition questions)** | **Choice FOR a dental treatment**   - **When you think about your experiences at the dentist in the past:**   What reasons did influence you when choosing a dental treatment?   - Do your experiences influence your future decision-making behavior?   - To what extent do the experiences influence your future decision-making behavior? - In case you have used a (financial) supporting measure, e.g., dental supplementary insurance/”Bonus booklet”: Have you been satisfied with these measures? | After approx. 1.5h duration: break and analysis of given statements on experiences:  Summarizing the given statements and transformation to **reasons** and forming **categories**.  Formulation of **expectations**, written on a paper card by participants and pinned on a chart board (visible for all participants). |
|  | **Choice AGAINST a dental treatment**   - **When you think about your experiences at the dentist in the past:**   What reasons did influence you when NOT choosing a dental treatment?  Was your decision based on whether you had to make a co-payment?   - Do your experiences influence your future decision-making behavior? - To what extent do the experiences influence your future decision-making behavior? |  |
| **VII Core questions: presentation of identified reasons and addition of further expectations (Key questions)** | - During the break, we have analyzed your statements about previous experiences, and we specified reasons, summarized it to categories, and formulated expectations. - Do you see your experiences and resulting expectations in our findings? - Do you miss a certain experience or expectation here?   - If yes, please name it! - What are further expectations that are important to you, that we have not heard in the interview yet? - **Independently from your previous experiences:**   - What are your expectations in dental care?   - What would be important to you? | Presentation of categories and reasons expressed as expectations. Support presentation of experiences and expectations with statement examples. |
| **VIII Concluding questions: selection of  “most important” reasons (Ending questions)** | - Discussion: Which of the presented reasons are most important to you? - I would like to ask you to take a moment and think about your choice. We would like to rank the reasons “most important” to you. Again: What are the three “most important” reasons to you? | Count number of choices in a **tally sheet** on the chart board (visible for all participants). |
| **IX End** | - This leads us to the end of our interview. - Are there any questions or comments from your side? - **We sincerely thank you** for your participation! | - |
