# Supplementary material for: Reasons for (not) choosing dental treatments—A qualitative study based on patients’ perspective
Source: PLoS One. 2022 May 25;17(5):e0267656. doi: 10.1371/journal.pone.0267656 (PMC9132305; doi:10.1371/journal.pone.0267656)
Supplement: S2 Table — (DOCX) [file pone.0267656.s013.docx]

**S2 Table. Coding-tree and coding scheme, including codebook.**

| **Coding-tree** (schematic)  *Code emerged in further course*  code, 1st version: no. …  code, 1st version: no.4  code, 1st version: no.3  code, 1st version: no.2  code, 1st version: no.1  category …  reason no.4  subcategory …  reason no.3  reason no. …  reason no.2  reason no.1  code, 2nd version: no. …  code, 2nd version: no.5  code, 2nd version: no.4  code, 2nd version: no.3  code, 2nd version: no.2  code, 2nd version: no.1  …  subcategory 1 | | | | | | | |
| --- | --- | --- | --- | --- | --- | --- | --- |
| **Coding scheme** | | | | | | | |
| **Codebook** | | | | | **Reasons** (codes of final version further refined) | **Subcategories** | **Categories** |
| **No.** | **1st version (defined after consensus of interview group 1)** | | **Final version (refined after consensus of interview group 1–4)** | |  |  |  |
|  | **Codes, 1st version** | **Description and researcher notes, 1st version** | **Codes, final version** | **Description and researcher notes, final version** |  |  |  |
| 1 | Complaints or pain before treatment | Pain or discomfort leading patient to treatment. | Complaints or pain before treatment | Pain or discomfort leading patient to treatment. | Current complaints | Preconditions | Health care service |
| 2 | Self-diagnosis/-initiative | Patient's decision is based on already formed opinion about her/his needs for treatment, e.g., asks for treatment on own initiative. | Self-diagnosis/-initiative | Patient's decision is based on already formed opinion about her/his need for treatment, and requests treatment proactively. | Self-diagnosis |  |  |
|  | Recommendation by family, circle of friends and acquaintances | Treatment or dentist is recommended by family, circle of friends and acquaintances. | Recommendation of the service (by family, circle of friends and acquaintances, or other sources) | Treatment or dentist is recommended by family, circle of friends and acquaintances. |  |  |  |
|  | Recommendation by health insurance company | Treatment or dentist is recommended by health insurance company. | Recommendation of products or treatments (by health insurance company, or other sources) | Treatment or dentist is recommended by health insurance company. |  |  |  |
|  | Recommendation by other sources (e.g., internet) | Treatment or dentist was recommended on internet-pages or -portals, and other sources, e.g., popularity according to other patients: "He's always fully booked.". |  |  |  |  |  |
| 3 | Constitution of patient (e.g., age) | Patient's physical condition, e.g., pre-existing disease, good or bad oral health, age, smoking. | Constitution of patient | Patient's physical condition, e.g., pre-existing disease, good or bad oral health, age, smoking. | Patient's constitution |  |  |
| 4 | Recommendation by dentist/staff | Treatment is recommended by dentist. | Recommendation by dentist/staff | Treatment is recommended by dentist. | Professional recommendation |  |  |
| 5 | Prevention and prophylaxis | Treatment is taken by patient to care for precaution on dental health. | Prevention and prophylaxis | Treatment is taken by patient to care for precaution on dental health, i.e., including aftercare. | Prevention |  |  |
| 6 | Duration of treatment | Duration of single treatment session or multiple sessions. | Duration of treatment | Time for patient to complete treatment, i.e., including ways to and from dental office, and duration of a single treatment session or multiple sessions. | Duration of treatment | Treatment |  |
| 7 | Complaints/pain during treatment | Pain or discomfort during treatment, and extent of treatment. | Complaints/pain during treatment | Pain or discomfort during treatment, and extent of treatment. | Complaints during treatment process |  |  |
| 8 | "Bonus booklet" | The "bonus booklet" or receiving a stamp in the booklet is driver for utilizing treatment. | "Bonus booklet" | The "bonus booklet" or receiving a stamp in the booklet is driver for utilizing treatment. | "Bonus booklet" | Costs |  |
| 9 | "Bonus program" | "Bonus program" of a specific health insurance company is driver for utilizing treatment. | "Bonus program" | "Bonus program" of a specific health insurance company is driver for utilizing treatment. | "Bonus program" |  |  |
| 10 | Co-payment by patient | Amount of money that must be paid by patient. | Co-payment by patient | Amount of money that must be paid by patient. | Out-of-pocket payment |  |  |
| 11 | Employment status of patient | Patient's decision is based on her/his employment situation, e.g., student. | Employment status of patient | Patient's decision is based on her/his employment situation, e.g., student. | Income |  |  |
| 12 | Cost-benefit ratio | Patient weighs costs against expected benefits before treatment start. | Cost-benefit ratio | Patient weighs costs against expected benefits before treatment start. | Cost-benefit |  |  |
|  | Cost-risk ratio | Patient weighs costs against expected risks before treatment start. |  |  |  |  |  |
| 13 | Health insurance membership | Health insurance affiliation results in benefits, e.g., in terms of costs when using a treatment or special dentist. | Health insurance affiliation | Health insurance affiliation results in benefits, e.g., in terms of costs when using a treatment or special dentist. | Insurance coverage |  |  |
| 14 | Possibility of installment payments | Dentist offers possibility to pay for treatment by installments. | Possibility of installment payments | Dentist offers possibility to pay for treatment by installments. | Installment |  |  |
| 15 | *Code emerged in further course* | | Actual costs not calculable | Dentist and the patient cannot predict final cost of treatment before start. | Actual costs |  |  |
| 16 | Supplementary insurance | Supplementary insurance results in reduction of costs for treatment. | Supplementary insurance | Supplementary insurance results in reduction of costs for treatment. | Dental supplementary insurance |  |  |
| 17 | Second offer | Second offer is obtained for treatment regarding costs. | Second offer | Second offer is obtained for treatment. | Second offer |  |  |
|  | Second opinion | Second opinion by another dentist, that is consulted regarding treatment. |  |  |  |  |  |
| 18 | Aesthetics | Patient uses service to maintain/achieve a certain aesthetic look. | Aesthetics | Patient uses service to achieve a certain level of aesthetics. | Aesthetics | Outcomes |  |
| 19 | Complaints/pain after treatment | Pain or discomfort after treatment. | Complaints/pain after treatment | Pain or discomfort after treatment. | Complaints after treatment |  |  |
| 20 | Durability of treatment result | Expected lifetime of treatment result, e.g., when does crown need to be renewed. | Durability | Expected lifetime of treatment result, e.g., when does crown need to be renewed. | Durability |  |  |
| 21 | Influence on health (e.g., non-compatibility) | Treatment may have an impact on health, e.g., causing allergy. | Influence on health | Presumed influence of treatment or product on health. | Influence on health |  |  |
| 22 | *Code emerged in further course* | | Tooth preservation (teeth) | Preservation of teeth is important to patient (e.g., no grinding of healthy teeth). | Functionality |  |  |
|  |  |  | Tooth preservation (dentition regarding functionality) | Preservation or restoration of teeth is important to patient for maintaining functionality. |  |  |  |
| 23 | Biological or holistic approach | Dentist offers treatment based on biological or holistic approaches. | Biological-holistic approach | Dentist offers treatment based on biological or holistic approaches. | Holism |  |  |
| 24 | *Code emerged in further course* | | Compatibility | Material or medication causes incompatibility response. | Compatibility |  |  |
| 25 | Dentist: Training | Studies and training of dentist. | Training and title | Academic title, degree, or training of the dentist, e.g., PhD, Prof, head of dental office. | Training | Professional skills | Dentist & dental office |
|  | Dentist: Academic title | Academic title or degree of dentist, e.g., PhD, Prof, head of dental office, is important for patient. |  |  |  |  |  |
|  | Specialist | Treatment is performed by a specialist. | Specialist | Treatment is performed by a specialist. |  |  |  |
| 26 | *Code emerged in further course* | | Appropriate advice | Dentist gives advise according to available treatment options in a way the patient can understand. | Adequate advice |  |  |
| 27 | Competence: Applying knowledge | Dentist is perceived as competent after treatment is done, e.g., treatment was well done or went smoothly. | Applying knowledge | Dentist is perceived as competent after treatment is done, e.g., treatment was well done or went smoothly. | Work experience |  |  |
|  | Competence: Maintaining knowledge | Dentist is perceived as competent in her/his profession, e.g., consultation is offered, saying "competence". | Maintaining knowledge | Dentist is perceived as competent in her/his profession, e.g., consultation is offered, saying "competence". |  |  |  |
|  | Dentist: Age | Age or experience of dentist. | Professional experience | Practical experience of dentist in her/his profession, i.e., also age of dentist. |  |  |  |
|  | *Code emerged in further course* | |  |  |  |  |  |
| 28 | Error | Dentist has made a mistake during previous treatment. | Error | Dentist has made a mistake during previous treatment. | Medical error |  |  |
| 29 | Sensitivity | Dentist is sensitive and can relate well to patient during treatment. | Adaptation of treatment | Dentist adjusts treatment to patient's physiological or psychological needs during treatment. Note: = Code “Dentist: Paying attention”. | Flexibility |  |  |
| 30 | Way of working: Accuracy | Dentist's working method is perceived as accurate, e.g., comprehensive diagnosis. | Accuracy | Dentist's way of working is perceived as accurate. | Accuracy |  |  |
| 31 | Interdisciplinarity | Various (dental) disciplines are represented in dental office. | Interdisciplinarity | Dentists and physicians of various (dental) disciplines are represented in dental office, or dental office is integrated in a medical center. | Interdisciplinarity |  |  |
|  | Group practice | Dental office is organized as a community of several dentists and physicians, e.g., availability of an oral surgeon. | Group practice expertise | Dental office is organized as a community of several dentists. |  |  |  |
| 32 | Information on treatment by dentist/staff | Patient feels (not) sufficiently informed/well advised by dentist regarding treatment procedure and outcome. | Information on treatment by dentist/staff | Patient feels (not) sufficiently informed/well advised by dentist regarding treatment procedure and outcome. | Professional treatment and costs information |  |  |
|  | Information on treatment costs by dentist/staff | Patient feels (not) sufficiently informed/well advised by dentist regarding cost of treatment. | Information on treatment costs by dentist/staff | Patient feels (not) sufficiently informed/well advised by dentist regarding cost of treatment. |  |  |  |
| 33 | Way of working: Calmness/composure | Dentist rests within her-/himself during treatment. | Calm way of working | Dentist rests within her-/himself before, during and after treatment. | Calmness |  |  |
| 34 | Dentist: Nationality | Nationality of dentist, e.g., resulting in language barrier. | No language barrier | No difficulties in transmitting information, e.g., due to the dentist's nationality. | Language barrier | Social skills |  |
| 35 | Presentation of alternatives | Dentist suggests several alternatives before treatment. | Presentation of alternatives | Dentist suggests several alternatives before treatment. | Presentation of alternatives |  |  |
| 36 | Obtrusiveness | Patient perceives dentist as pushy or feels pressured to decide, e.g., time to decide/consider. | No obtrusiveness | Patient does not perceive dentist as intrusive / does not feel pressured to decide, e.g., time to decide. | Obtrusiveness |  |  |
| 37 | Dentist: Appearance | Look of dentist. | Appearance | Look of dentist. | Appearance |  |  |
| 38 | Courtesy/friendliness | Dentist and staff are perceived as friendly/courteous. | Courtesy/friendliness | Dentist and staff are perceived as friendly/courteous. | Courtesy/ friendliness |  |  |
|  | Respect for patient | Dentist behaves respectfully towards patient and gives undivided attention, patient feels taken seriously, and dentist takes time; negative example: dentist behaves violent towards patient. | Respect | Dentist behaves respectfully towards patient and staff. |  |  |  |
|  | Respect for staff | Dentist behaves respectfully towards staff. |  |  |  |  |  |
| 39 | Insight of dentist | Dentist's ability to make concessions and admit mistakes, and thus adjust treatment; with an influence on assessment of competence. | Critical faculty | Dentist's ability to make concessions and admit mistakes. Note: = Code “Dentist: Critical faculty”. | Ability to take  criticism |  |  |
| 40 | Consideration of patient's opinion | Patient feels involved in decision-making by dentist when taking her/his opinion into account, e.g., dentist changes opinion according to patient's wish. | Consideration of patient's opinion | Patient feels involved in decision-making by dentist when taking her/his opinion into account. | Patient opinion |  |  |
| 41 | Profit orientation | Dentist acts in her/his interest regarding profit. | Profit orientation | Dentist acts in her/his interest regarding profit. | Profit orientation |  |  |
| 42 | Seriousness | Dentist is perceived as serious, i.e., realization of confidentiality and medical secrecy. | Seriousness | Dentist is perceived as serious, i.e., realization of confidentiality and medical secrecy. | Seriousness |  |  |
| 43 | Trust in dentist | Patient trusts dentist; negative example: the dentist extends the treatment without discussion, dentist seems to not have a plan on treatment or be trustworthy, and patient feels helplessly to dentist during treatment. | Relationship of trust and trustworthiness | Patient trusts dentist. | Trust |  |  |
|  | Credibility | Dentist changes her/his mind about treatment. | Credibility | Dentist is not credible in her/his statements. |  |  |  |
|  | Self-confidence of dentist | Dentist is perceived as too (little) self-confident, i.e., insecurity or reticence. | Self-confidence | Dentist is perceived as self-confident. |  |  |  |
|  | Many years with a dentist | Patient has been with a dentist for many years and dentist knows the patient well, including course of disease. | Sympathy | Patient feels sympathy with dentist, and no interaction must have taken place beforehand, i.e., affection without reason. |  |  |  |
|  | *Code emerged in further course* | |  |  |  |  |  |
| 44 | *Code emerged in further course* | | Dentist takes time | Dentist adjusts time required before, during and after treatment to patient's individual circumstances. | Dentist takes time |  |  |
| 45 | Interhuman relations of dentist/staff | Characteristics of interaction between dentist and patient before, between, or after treatment; including interaction with children. | Interhuman relations | Characteristics of patient-oriented interaction of dentist before and after treatment. | Interhuman  relations |  |  |
| 46 | Equipment: medical staff (not dentist) | Patient's decision is based on medical staff, e.g., assistants and their characteristics; and number, appearance, gender, or competence. | Medical staff (not dentist) | Presence of medical staff in dental office, e.g., assistants and their characteristics. | Medical staff (not dentist) | Office staff & equipment |  |
| 47 | Equipment: medical (e.g., devices, materials) | Patient's decision is based on range of equipment available. | Medical-technical equipment | Availability of medical-technical equipment in dental office. | Medical-technical equipment |  |  |
|  | Innovation | Treatment is explicitly described as innovative or "up-to-date". | Innovation | Treatment is explicitly described as innovative or "up-to-date". |  |  |  |
| 48 | Equipment: non-medical (e.g., furniture) | Patient's decision is based on offer of non-medical equipment, e.g., waiting-room furniture, magazines, and drinks. | Non-medical equipment | Presence of non-medical equipment and conditions in dental office, e.g., waiting-room furniture, magazines, and drinks. | Non-medical equipment |  |  |
| 49 | Equipment: non-medical staff | Patient's decision is based on medical staff, e.g., receptionists and their characteristics. | Non-medical staff | Patient's decision is based on medical staff, e.g., receptionists and their characteristics. | Non-medical staff |  |  |
| 50 | Organization | Dental office processes are perceived as unorganized, e.g., missed appointments, unprepared dentist. | Coordination of dental office processes | Dental office processes are perceived as unorganized, e.g., missed appointments, unprepared dentist. | Coordination | Office processes |  |
| 51 | Hygiene | Treatment takes place under hygienic conditions. Note: ≠ Code "Cleanliness". | Hygiene | Treatment takes place under hygienic conditions. Note: ≠ Code "Cleanliness". | Hygiene |  |  |
|  | Cleanliness | Dental office rooms and location (e.g., staircase) are perceived as clean by patient. | Cleanliness | Dental office rooms and location (e.g., staircase) are perceived as clean by patient. |  |  |  |
| 52 | Service | Services perceived as exceptional, e.g., childcare, reminder postcards. | Measures of customer loyalty | Service offered by dental office, e.g., reminder calls, extended opening hours, childcare. | Patient orientation |  |  |
| 53 | Waiting time for appointment | Waiting time for appointment. | Waiting time | Waiting time in dental office in waiting-room (having appointment), right before treatment, or during treatment procedure. | Waiting time |  |  |
|  | Waiting time in dental office | Waiting time in dental office in waiting-room (having or not having appointment), right before treatment, or during treatment procedure. |  |  |  |  |  |
