# Supplementary material for: Reasons for (not) choosing dental treatments—A qualitative study based on patients’ perspective
Source: PLoS One. 2022 May 25;17(5):e0267656. doi: 10.1371/journal.pone.0267656 (PMC9132305; doi:10.1371/journal.pone.0267656)
Supplement: S3 Table — (DOCX) [file pone.0267656.s014.docx]

**S3 Table. Definitions of category “health care service”, its subcategories and reasons (n=24, no.1–24).**

| **Health care service:** Scope of using a treatment at dentist’s office, ranging from given preconditions, about actual treatment, and its costs, to outcomes. | | |
| --- | --- | --- |
| **Subcategories** | **No.** | **Reasons**: Definitions |
| **Preconditions:** Conditions given for patients considering an appointment at the dentist. | 1 | **Current complaints**: Pain or physical exertion before the first visit at the dentist’s office. |
|  | 2 | **Prevention**: Measures used regularly aiming at early detection and prevention of diseases. |
|  | 3 | **Patient's constitution**: Characteristics of patients’ physical conditions and state of health, e.g., pre-existing diseases, allergies. |
|  | 4 | **Professional recommendation**: Dentist’s advocacy for treatments after making a diagnosis. |
|  | 5 | **Self-diagnosis**: Patients’ decision-making based on own opinions, also using non-professional information-sources, e.g., internet, family, and friends. |
| **Treatment:** Diagnosis and therapy (prevention, tooth preservation and dentures). | 6 | **Complaints during treatment process**: Pain or physical exertion of patients during treatment. |
|  | 7 | **Duration of treatment**: Temporal efforts arising for patients during treatment process. |
| **Costs:** Financial burden for patients resulting from treatments and influencing reasons. | 8 | **Actual costs**: Overall financial expenditures for treatments, composed of calculable and incalculable costs, taken by single patient. |
|  | 9 | **Out-of-pocket payment**: Amount of co-payment for treatments taken by single patient. |
|  | 10 | **Income**: Financial earnings of single persons depending on employment status. |
|  | 11 | **Cost-benefit**: Assumed relation of cost and expected outcomes. |
|  | 12 | **Insurance coverage**: Overall or partial coverage of treatment costs by health insurance. |
|  | 13 | **"Bonus booklet"**: Incentive instrument of the German statutory health insurance (SHI). Paper booklet documenting annual dental check-ups, confirmed by stamp. SHI subsidies for dentures may increase with regular check-up in the last years before treatment. |
|  | 14 | **"Bonus program"**: Incentive instrument of the SHI designed individually by insurances. Members can voluntarily sign into those programs. When using defined prevention measures, e.g., health promoting activities or (dental-)medical check-ups, participants receive financial or non-financial bonuses. |
|  | 15 | **Dental supplementary insurance**: Private insurance in addition to existing insurance membership. Depending on the scope of services, it reduces the amount of out-of-pocket payments for certain treatments and expands treatment-choices. |
|  | 16 | **Second offer**: Patients may request a second opinion for planned treatment and its costs offered by another dentist. |
|  | 17 | **Installment**: Settlement of co-payments in staggered, small amounts. |
| **Outcomes:** Consequences of treatments for patients. | 18 | **Aesthetics**: Individual results of treatments perceived as beautiful. |
|  | 19 | **Compatibility**: Dental materials or drugs used in the treatment process do not induce any immune response, e.g., allergic and local toxic reactions. |
|  | 20 | **Durability**: Expected time from completion of one treatment to another medically or technically necessary treatment for same concern. |
|  | 21 | **Functionality**: Single teeth or dentition fulfill its natural purposes after treatment. |
|  | 22 | **Influence on health**: Effects of treatments on oral health and overall health of patients. |
|  | 23 | **Holism**: Approach considering whole person in treatments for overall health and consequently (oral) health. |
|  | 24 | **Complaints after treatment**: Pain or physical exertion of patients after last visit to dentist. |
