# Supplementary material for: Reasons for (not) choosing dental treatments—A qualitative study based on patients’ perspective
Source: PLoS One. 2022 May 25;17(5):e0267656. doi: 10.1371/journal.pone.0267656 (PMC9132305; doi:10.1371/journal.pone.0267656)
Supplement: S4 Table — (DOCX) [file pone.0267656.s015.docx]

**S4 Table. Definitions of category “dentist & dental office”, its subcategories and reasons (n=29, no.25–53).**

| **Dentist:** Person, who keeps degree in dentistry and examines patients on oral health and treats in case of diseases. **Dental office:** Location, where dental treatments and organizational aspects are performed. | | |
| --- | --- | --- |
| **Subcategories** | **No.** | **Reasons**: Definitions |
| **Professional skills:** Practical and theoretical competences of dentist in realization of treatments. | 25 | **Training**: Licensed qualifications of dentists, specializations, or academic degrees |
|  | 26 | **Work experience**: Professional experience of dentists. |
|  | 27 | **Medical error**: Treatment leads to unplanned and unwanted negative events or outcomes. |
|  | 28 | **Accuracy**: Conscientiousness and care of dentist while performing services. |
|  | 29 | **Calmness**: Reassuring and smooth work of dentist. |
|  | 30 | **Flexibility**: Possibility of treatment adjustments due to unplanned physiological or psychological needs of patients. |
|  | 31 | **Interdisciplinarity**: Additional (dental-)medical expertise within the dental office (e.g., group practice) or in the surrounding area (e.g., medical center). |
|  | 32 | **Professional treatment and costs information**: Dentist provides complete information to patients about treatments and related costs. |
|  | 33 | **Adequate advice**: Dentist presents treatment alternatives to patients in a way they can understand. |
|  | 34 | **Language barrier**: Difficulties in verbal communication between dentist, staff, and patients due to different languages. |
| **Social skills:** Set of dentist's abilities in communication and interaction, allowing to respond to patients’ needs. | 35 | **Interhuman relations**: Verbal and non-verbal communication between dentist and patients, perceived as pleasant. |
|  | 36 | **Trust**: Bilateral trust-based relationship between dentist and patients evolving from past experiences. |
|  | 37 | **Dentist takes time**: Temporal effort of dentist is adjusted to individual needs of patients before, during and after treatments. |
|  | 38 | **Courtesy/friendliness**: Respectful and considerate behavior between dentist, staff, and patients. |
|  | 39 | **Ability to take criticism**: Assessment of dentist's work by patient is accepted by the dentist. |
|  | 40 | **Patient opinion**: Consideration of patients’ perspective and needs in a treatment. |
|  | 41 | **Profit orientation**: Dentist’s behavior seems to gear towards an economic gain. |
|  | 42 | **Obtrusiveness**: Dentist and staff attempts directing patients to certain treatment decisions, e.g., not giving enough time to rethink. |
|  | 43 | **Presentation of alternatives**: Dentist gives patients an overview of available possibilities of treatment. |
|  | 44 | **Seriousness**: Trustworthy handling of patients’ data, e.g., regarding data security, and costs transparency. |
|  | 45 | **Appearance**: Look of dentist and staff, e.g., look of teeth and clothes. |
| **Office staff & equipment:** Sum of human resources, dental materials, medical and non-medical units in dental office. | 46 | **Medical staff (not dentist)**: Personnel with (dental-)medical education, e.g., dental assistant. |
|  | 47 | **Non-medical staff**: Personnel without (dental-)medical education, e.g., appointment coordinator. |
|  | 48 | **Medical-technical equipment**: Equipment, instruments, and apparatus for (dental-)medical purposes, e.g., dental chair, drills. |
|  | 49 | **Non-medical equipment**: Equipment and objects not for (dental-)medical purposes, e.g., furnishing, magazines and drinks in waiting room. |
| **Office processes:** Processes for realization of treatments. | 50 | **Patient orientation**: Services addressing non-medical needs of patients, e.g., appointment reminder by postal mail and mobile messaging, appointment offers outside opening hours, on-site childcare. |
|  | 51 | **Coordination**: Correct flow of processes. Treatments can be realized in time and as planned. |
|  | 52 | **Waiting time**: Duration of time to appointment, in the waiting room and in the dental office (before and during treatments). |
|  | 53 | **Hygiene**: Given medical sterility of equipment, instruments and apparatus used for treatments. |
