# Supplementary material for: Reasons for (not) choosing dental treatments—A qualitative study based on patients’ perspective
Source: PLoS One. 2022 May 25;17(5):e0267656. doi: 10.1371/journal.pone.0267656 (PMC9132305; doi:10.1371/journal.pone.0267656)
Supplement: S5 Table — (DOCX) [file pone.0267656.s016.docx]

**S5 Table. Additional statements on patients’ reasons for (not) choosing a dental treatment.**

| **Categories and subcategories** | | **Reasons** | **Statements** |
| --- | --- | --- | --- |
| Health care service | Preconditions | Professional recommendation | [The dentist] advised me, that the tooth next to it, a crown- and root-treated one, should also be extracted. Because on the radiograph you could see that it was somehow chronically inflamed. (group 2, #1) |
|  |  | Self-diagnosis | I decided against the treatment because it is not yet so acute. I don't have the feeling that I need that. (group 1, #3) // Then I started to do my own research and somehow read myself into the topic and decided what is useful, what is not useful. (group 4, #5) |
|  | Outcomes | Functionality | I had decided for an implant about eight years ago, after advice of dentist […]. The molars had to be extracted. For those an implant was recommended to me. And then it was implanted, which was one and a half molar. So, I had the chance to chew. (group 3, #9) |
|  |  | Complaints after treatment | I was not that happy with the treatment. Because the anesthesia was too strong, I felt it for days afterwards, somehow. (group 1, #7) // Even if you have chosen a dentist and stayed with him, you look by yourself how the tooth feels. And if there are any problems after three, four, five months, then you will look for a new dentist. (group 1, #1) |
| Dentist & dental office | Professional skills | Medical error | [The dentist] unfortunately did not diagnose this decline in the gum for years. If someone had said that earlier, I might have been able to do something earlier. That annoys me a little. (group 1, #6) // I noticed it and thought “Wait a minute!” and raised my hand, because [the dentist] was about to drill the tooth. I said, “We are in a surgery on the wrong tooth!”. (group 4, #4) // When inserting the dental crown, it was not properly cleaned and disinfected. […] And now, within less than two years, the tooth got rotten underneath. And then I lost my whole tooth. That was not a very nice experience. (group 4, #3) |
|  |  | Professional treatment and costs information | There is a lack of transparency regarding fees. I would like to see the scale of charges, that counts for everyone and not that every single dental office creates it for itself.” (group 2, #4) |
|  | Social skills | Interhuman relations | When you enter the dental office and [the dentist] looks into your eyes. First, he really asks you how you're doing and how it's going. Nothing [for the treatment] was done at first consultation. (group 1, #2) |
|  |  | Courtesy/friendliness | It was so unpleasant painfully and I started to cry. And then they said, “What is your concern?”. Of course, you will not go there again, right? I think that was very tough. (group 3, #5) |
|  |  | Profit orientation | In contrast to my gynecologist, who I have had for 41 years, I changed dentists quite often until now. I am with someone, where I am really satisfied with, now. He always gives me the right advice and does not only look at money. For example, he does not always suggest some complete self-payment treatment. (group 2, #5) // The dentist said [that treatment] would not bring him any money. […] These 20€ are too little for him. Because his Porsche just eats more. Excuse me, this is annoying. (group 4, #2) |
|  |  | Obtrusiveness | It happened five minutes before surgery. Imagine, I have already gotten my anesthesia injection and then they show me “Please sign that [contract]”. And I said “What is it? What should I sign here?”. (group 1, #1) // [The dentist] informed me about treatment options and gave me a corresponding estimate of costs, and time to think about it. (group 1, #3) |
|  |  | Presentation of alternatives | This is what I name a spectrum, which means, if there is a problem, that from one end to the other end I am explained exactly what [treatment] is possible and what are alternatives. If I have a dentist, who takes part in this discussion, then I can make a better decision. (group 4, #5) |
|  | Office staff & equipment | Medical-technical equipment | I look at the devices. [...] For example, […] they put a screen right in front of my eyes, where I can see every single second. He has such a small camera and takes pictures of every phase of treatment and of what is done. Quality of photography really is super nice. I might not have seen my teeth like I did on this screen. [The dentist] also works with a microscope, instead of glasses. It is such a big microscope. And that's a sign for me. (group 1, #1) |
|  |  | Non-medical equipment | The whole dental office. How does it look like? Is there something to drink, for example, or how do you sit [in the waiting room]. So, I quit going to dentist appointments when I must wait for a long time uncomfortably. (group 3, #5) |
|  | Office processes | Coordination | I sit [in the waiting room] for two or 2.5 hours and then finally sit on the dental chair. “What did we plan to do today? Oh yes, well.”. And then [the dentist] digs into his drawer and says “Well, let us call the laboratory again.”, and then the dental assistant calls the laboratory, and it turns out, [my tooth model] has not arrived yet. (group 4, #4) |
|  |  | Hygiene | When a dental assistant comes into [the dental office] and uses equipment that is meant to be touched by dentist only. I think that is unhygienic, because another person has touched it. (group 3, #6) |
| (no. of interview group, participant #); // next statement. | | | |
