# Supplementary material for: Reasons for (not) choosing dental treatments—A qualitative study based on patients’ perspective
Source: PLoS One. 2022 May 25;17(5):e0267656. doi: 10.1371/journal.pone.0267656 (PMC9132305; doi:10.1371/journal.pone.0267656)
Supplement: S6 Table — (DOCX) [file pone.0267656.s017.docx]

**S6 Table. Distributions of sorted statements on reasons.**

| Category | Subcategory | No. | Reason | Statements on reasons | | "Unclear" statements |
| --- | --- | --- | --- | --- | --- | --- |
|  |  |  |  | for choosing dental treatments | for NOT choosing dental treatments |  |
| Health care service | Preconditions | 1 | Current complaints |  |  | x |
|  |  | 2 | Self-diagnosis |  |  | x |
|  |  | 3 | Patient's constitution |  |  | x |
|  |  | 4 | Professional recommendation |  |  | x |
|  |  | 5 | Prevention |  |  | - |
|  | Treatment | 6 | Duration of treatment |  |  | x |
|  |  | 7 | Complaints during treatment process |  |  | x |
|  | Costs | 8 | **"Bonus booklet"** |  |  | - |
|  |  | 9 | **"Bonus program"** |  |  | - |
|  |  | 10 | Out-of-pocket payment |  |  | x |
|  |  | 11 | Income |  |  | x |
|  |  | 12 | Cost-benefit |  |  | - |
|  |  | 13 | Insurance coverage |  |  | - |
|  |  | 14 | Installment |  |  | - |
|  |  | 15 | Actual costs |  |  | - |
|  |  | 16 | Dental supplementary insurance |  |  | - |
|  |  | 17 | Second offer |  |  | x |
|  | Outcomes | 18 | Aesthetics |  |  | x |
|  |  | 19 | Complaints after treatment |  |  | - |
|  |  | 20 | Durability |  |  | - |
|  |  | 21 | Influence on health |  |  | - |
|  |  | 22 | Functionality |  |  | x |
|  |  | 23 | Holism |  |  | - |
|  |  | 24 | Compatibility |  |  | - |
| Dentist & dental office | Professional skills | 25 | Training |  |  | x |
|  |  | 26 | **Adequate advice** |  |  | - |
|  |  | 27 | Work experience |  |  | x |
|  |  | 28 | **Medical error** |  |  | x |
|  |  | 29 | Flexibility |  |  | x |
|  |  | 30 | Accuracy |  |  | - |
|  |  | 31 | Interdisciplinarity |  |  | x |
|  |  | 32 | Professional treatment and costs information |  |  | x |
|  |  | 33 | Calmness |  |  | - |
|  | Social skills | 34 | Language barrier |  |  | - |
|  |  | 35 | Presentation of alternatives |  |  | - |
|  |  | 36 | Obtrusiveness |  |  | x |
|  |  | 37 | Appearance |  |  | - |
|  |  | 38 | Courtesy / friendliness |  |  | x |
|  |  | 39 | **Ability to take criticism** |  |  | - |
|  |  | 40 | Patient opinion |  |  | x |
|  |  | 41 | Profit orientation |  |  | x |
|  |  | 42 | Seriousness |  |  | - |
|  |  | 43 | Trust |  |  | x |
|  |  | 44 | Dentist takes time |  |  | - |
|  |  | 45 | Interhuman relations |  |  | x |
|  | Office staff & equipment | 46 | Medical staff (not dentist) |  |  | x |
|  |  | 47 | Medical-technical equipment |  |  | x |
|  |  | 48 | Non-medical equipment |  |  | x |
|  |  | 49 | **Non-medical staff** |  |  | - |
|  | Office processes | 50 | **Coordination** |  |  | - |
|  |  | 51 | Hygiene |  |  | - |
|  |  | 52 | Patient orientation |  |  | - |
|  |  | 53 | Waiting time |  |  | x |

**Reason** in bold letters – all statements refer either to reasons for choosing or NOT choosing dental treatments,


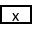
“unclear” statement(s): it remains unclear whether the statements by participants refer to choosing or NOT choosing dental treatments,


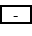
no “unclear” statement,


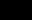
more than 50% of statements belong to reasons for (NOT) choosing dental treatments,


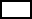
 less than 50% of statements belong to reasons for (NOT) choosing dental treatments, and


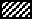
 statements for choosing and NOT choosing dental treatments are distributed equally (50/50%).
