# Supplementary material for: Reasons for (not) choosing dental treatments—A qualitative study based on patients’ perspective
Source: PLoS One. 2022 May 25;17(5):e0267656. doi: 10.1371/journal.pone.0267656 (PMC9132305; doi:10.1371/journal.pone.0267656)
Supplement: Attachment — Submitted filename: Response to Reviewers.docx [file pone.0267656.s018.docx]

Dear Dr. Hüttig,

First and foremost, we would like to thank you and your team for providing us with the opportunity to revise the manuscript. We have taken care to consider the reviewers’ concerns carefully, addressing each in as straightforward a manner as possible. We found their comments to be insightful and helpful and feel that the manuscript has benefited greatly as a result.

Below please find the responds to each point raised by the academic editor and both reviewers.

We included a marked-up copy of our manuscript that highlights changes made to the original version 'Revised Manuscript with Track Changes' as well as an unmarked version of our revised manuscript ('Manuscript'). We have also added further documents as supporting information.

Please do not hesitate to contact us should anything remain unclear, or if any further revisions are needed.

Thank you, again, for your time and consideration!

Sincerely,

Susanne Felgner

**Editor’s comments**

*“In case of a) ([a major revision]) you and your COs have to rework the data and rewrite the paper completely regarding the points raised by both reviewers. This encompasses a clear explanation of the limitations due to participant selection (bias) of your study by age and location (Berlin is not representative for Germany. Patients in Stuttgart area are different in their oral health behavior, decision, and willingness to pay compared to Berlin). You should consult more literature and a dentist with a German license to get more insight to the system. For instance, "out of the pocket" payments are applicable for treatments which are not standard of the SHI (BEMA catalog) and in case of dentures for patients not in social support. Last mentioned will get the SHI-based denture free of charge. Sometimes these options might not be offered to the patients due to cost-benefit-calculations of the dental practice - which is a violation of law. Some of these aspects can be found i.e. in Behrend & Huettig 2016 as well as findings from patient interviews in Florian M. Behrends thesis (available as PDF via Uni Tübingen). You can find more literature there - even if the amount is limited for Germany still today.”*

Thank you very much for your helpful comments and suggestions. We reworked the data and rewrote huge parts of the paper according to the points raised by the reviewers. We additionally analyzed the data according to the reviewer’s (#2) suggestions.

We fully agree that the limitations regarding the selection of participants need to be clearly explained in the manuscript. For our study, we exclusively recruited participants aged ≥ 18. For organizational reasons, we conducted the interviews at the Technische Universität Berlin. Therefore, the recruitment strategy was limited to Berlin locations and media, except for the advertisement on Facebook and eBay. Being the study’s analyzing researchers, we do not know the actual participants’ residence. We also did not request the postal codes via the questionnaire that participants filled in. The limitations section now includes a more critical view on representativeness of the results. We indicate that certain behaviors concerning dental treatments (e.g., oral health behavior, decision-making, or willingness-to-pay) can be region-specific and may lead to bias.

We assumed we had clearly presented the regulations of co-payment within the framework of the SHI-system, with a focus on dentures (e.g., “Although many basic treatments such as dental check-ups and tooth-fillings [16] are covered fully by SHI, treatments exceeding standard care services as well as dentures are only covered in parts [17].”; p.4 in the manuscript). Of course, we made revisions and additions to the manuscript that contribute to readers’ understanding. For example, we now clearly explain the exception mentioned in the Social Code Book V by adding information on up to 100%-coverage for patients fulfilling certain criteria (e.g., patients with low income, or receiving welfare beneficiaries; p.5 in the manuscript). We also added an arrow to Fig 1 representing the scope of SHI-payment. This arrow depicts the 100% SHI-payment for dentures.

**Journal Requirements**

*“1. Please ensure that your manuscript meets PLOS ONE's style requirements, including those for file naming. The PLOS ONE style templates can be found at* [*https://journals.plos.org/plosone/s/file?id=wjVg/PLOSOne_formatting_sample_main_body.pdf*](https://journals.plos.org/plosone/s/file?id=wjVg/PLOSOne_formatting_sample_main_body.pdf) *and* [*https://journals.plos.org/plosone/s/file?id=ba62/PLOSOne_formatting_ sample_title_authors_affiliations.pdf*](https://journals.plos.org/plosone/s/file?id=ba62/PLOSOne_formatting_%20sample_title_authors_affiliations.pdf)*.”*

According to the PLOS ONE style templates, we changed the file naming of figures (e.g., “Fig1.tiff”) and the supporting information (e.g., “S1_Table.docx”). We also changed the citation of figures (e.g., “Fig 1”), tables (e.g., “Table 1”) and supporting information (e.g., “S1 Table”) in the manuscript, and the table titles in the supporting information documents. The article title is now written in non-bold letters.

*“2. When reporting the results of qualitative research, we suggest consulting the COREQ guidelines:* [*http://intqhc.oxfordjournals.org/content/19/6/349*](http://intqhc.oxfordjournals.org/content/19/6/349)*. In this case, please consider including more information on the interviewers, their training and characteristics; and please provide the interview guide used.”*

We now follow the criteria of the COREQ checklist. Accordingly, we added information to respective sections (e.g., methods section). We also used the COREQ checklist for explicit and comprehensive reporting of our qualitative study. We added the table to the supporting information (S1 File). In this respect, we have taken the opportunity to give more information about the interviewers/researchers. For example, we added additional information of the interviewers (SF, CH) on their experiences in conducting and analyzing qualitative studies [e.g., “Provision and financing of assistive technology devices in Germany: A bureaucratic odyssey? The case of amyotrophic lateral sclerosis and Duchenne muscular dystrophy.” by Henschke (2012), and “Physicians' Decision Making on Adoption of New Technologies and Role of Coverage with Evidence Development: A Qualitative Study.” by Felgner, Ex & Henschke (2018)]. In addition, SF and CH have been teaching qualitative research methods at the university. Researcher MB was also involved in the interviews as an assistant (responsible for recording). Later she also analyzed the interviews as employed research fellow. In the methods section, we added characteristics of the moderator, co-moderator, and assistants, e.g., sex, and positions at the department. Further, we added the interview guide to the supporting information (S1 Table).

*“3. We note that you have indicated that data from this study are available upon request. PLOS only allows data to be available upon request if there are legal or ethical restrictions on sharing data publicly. For information on unacceptable data access restrictions, please see* [*http://journals.plos.org/plosone/s/data-availability#loc-unacceptable-data-access-restrictions*](http://journals.plos.org/plosone/s/data-availability#loc-unacceptable-data-access-restrictions)*. In your revised cover letter, please address the following prompts:*

*a) If there are ethical or legal restrictions on sharing a de-identified data set, please explain them in detail (e.g., data contain potentially identifying or sensitive patient information) and who has imposed them (e.g., an ethics committee). Please also provide contact information for a data access committee, ethics committee, or other institutional body to which data requests may be sent.*

*b) If there are no restrictions, please upload the minimal anonymized data set necessary to replicate your study findings as either Supporting Information files or to a stable, public repository and provide us with the relevant URLs, DOIs, or accession numbers. Please see* [*http://www.bmj.com/content/340/bmj.c181.long*](http://www.bmj.com/content/340/bmj.c181.long) *for guidelines on how to de-identify and prepare clinical data for publication. For a list of acceptable repositories, please see* [*http://journals.plos.org/plosone/s/data-availability#loc-recommended-repositories*](http://journals.plos.org/plosone/s/data-availability#loc-recommended-repositories)*. We will update your Data Availability statement on your behalf to reflect the information you provide.”*

Thank you for calling this important point to our attention. Before conducting the interviews, an ethics application was submitted to, and approved by the ethical committee of the Technische Universität Berlin. The application addresses the use of collected data: by signing the informed consent, interview participants confirmed that anonymized quotes based on the anonymized transcripts could be used for publication as part of the research project. In compliance with this requirement, we give single statements of the interview participants in the manuscript (Table 2, p.13; Fig 2) and the supporting information (S8, S9 and S10 Files, and S5 Table) for traceability and support of our study findings. We do not offer access to anonymized interview transcripts in another format, extend, or on demand. We have updated our Data Availability statement regarding this information.

According to the informed consent personal data of the participants is only presented in aggregated form so that no conclusions can be drawn about individual persons. For this reason, we do not provide the data set on the results of the survey on socio-demographic characteristics by questionnaire.

We have also attached the participants’ information document, the informed consent, and the participants questionnaire on socioeconomic characteristics as supporting information to the manuscript (S2, S3, and S4 Files).

Contact information of the ethical committee is:

Ethical committee of the Department of Psychology and Ergonomics, Technische Universität Berlin; Secretary MAR 3-2, Marchstraße 23, 10587 Berlin, Germany; phone: 049/03031422370, email: ethik@ipa.tu-berlin.de, chairman: Dr. Stefan Brandenburg.

**Reviewers’ comments**

*“1. Is the manuscript technically sound, and do the data support the conclusions? The manuscript must describe a technically sound piece of scientific research with data that supports the conclusions. Experiments must have been conducted rigorously, with appropriate controls, replication, and sample sizes. The conclusions must be drawn appropriately based on the data presented. Reviewer #1: Partly[,] Reviewer #2: Partly”*

Thank you very much for the assessment. We hope to meet the mentioned criteria even better after revising and making additions to the manuscript according to the following comments.

*“2. Has the statistical analysis been performed appropriately and rigorously? Reviewer #1: Yes[,] Reviewer #2: N/A”*

*“3. Have the authors made all data underlying the findings in their manuscript fully available? The PLOS Data policy requires authors to make all data underlying the findings described in their manuscript fully available without restriction, with rare exception (please refer to the Data Availability Statement in the manuscript PDF file). The data should be provided as part of the manuscript or its supporting information, or deposited to a public repository. For example, in addition to summary statistics, the data points behind means, medians and variance measures should be available. If there are restrictions on publicly sharing data—e.g. participant privacy or use of data from a third party—those must be specified. Reviewer #1: Yes[,] Reviewer #2: No”*

Thank you for the assessment. Before conducting the interviews, an ethics application was submitted to, and approved by the ethical committee of the Technische Universität Berlin. The application addresses the use of collected data: by signing the informed consent, interview participants confirmed that anonymized quotes based on the anonymized transcripts could be used for publication as part of the research project. In compliance with this requirement, we give single statements of the interview participants in the manuscript (Table 2, p.13; Fig 2) and the supporting information (S8, S9 and S10 Files, and S5 Table) for traceability and support of our study findings. We are not allowed to offer access to anonymized interview transcripts in another format, extend, or on demand. According to the informed consent personal data of the participants is only presented in aggregated form so that no conclusions can be drawn about individual persons. For this reason, we do not provide the data set on the results of the survey on socio-demographic characteristics by questionnaire.

*“4. Is the manuscript presented in an intelligible fashion and written in standard English? PLOS ONE does not copyedit accepted manuscripts, so the language in submitted articles must be clear, correct, and unambiguous. Any typographical or grammatical errors should be corrected at revision, so please note any specific errors here. Reviewer #1: Yes[,] Reviewer #2: No”*

We have proof-read the manuscript again and corrected remaining typographical and grammatical errors.

**Reviewer #1:**

*“The authors present a study about reasons of choice in dental care treatment. In Germany, every adult visits the dentist 2.4 times a year on average. In addition to paid preventive care, there are four reasons for this: pain, functional limitations, appearance and psychosocial impairments. Many older people have dentures (that is good). Sometimes over-treatment takes place (that is bad). For some groups in the population, however, there are barriers to using dental care: children, the very old, the disabled, multimorbid (ASA 3+), pain or fear patients etc. For these groups we need qualitative studies in order to break down the barriers but not for healthy middle-aged women to increase the risk of over-treatment. The topic and the methodology of the paper are ok, only the target group is unsuitable in the opinion of the reviewer.”*

We fully agree that especially those patient subgroups you mentioned should be part of qualitative studies. However, we think that it is also important to know what criteria are used by patients when deciding for or against a dental treatment. Even if there is full coverage for basic dental care and partly coverage for dentures (basic dentures, defined by the Federal Joint Committee), some treatments in dental care and especially dentures can be very expensive. Additionally, patients have the choice between different materials and treatments. Compared to inpatient care for instance, patients have high out-of-pocket payments. In inpatient care physicians finally decide on the product/procedure. Regarding dentures there are different opportunities for patients (e.g., implant vs. bridge). Physicians may suggest a treatment, but the final decision is taken by patient and may have different reasons: e.g. financial burden, risk of procedures etc. To our knowledge there is no study investigating those reasons. Therefore, the aim of our study was to identify patients’ reasons for choosing or not choosing a dental treatment, following an inductive approach and without a restriction to patients with special needs or diseases. However as mentioned, we fully agree that dental care of patient subgroups (e.g., children, patients with depression disease) should be part of future research. Thank you for this important hint.

**Reviewer #2:**

*1 Summary of the research*

*“The main research question of the current manuscript is twofold: (a) What factors influence patients in Germany in their decision on dental treatment? (b) How do patients assess cost coverage of dental services by German statutory health insurance? The authors claim to have identified (a) themes affecting patients’ decision-making on dental treatment and (b) financial challenges of dental treatments for the patients and ways how they take action against them. The authors draw the following conclusions: There are different reasons for choosing or not choosing dental treatment which can be categorised hierarchically into two main categories with several sub-categories each. Furthermore, financial aspects play a major role in decision-making for or against dental treatment and patients employ different measures to alleviate this financial burden. One strength of this study is that the research question is probably of interest for many different audiences, e. g. researches, practitioners, policy makers, and patients alike. Moreover, the approach is suitable for identifying (until today) unknown reasons for (not) choosing dental treatment. Finally it is to be welcomed that the authors used member checking as a quality assurance measure, i. e. they asked the participants after a break to confirm, reject, and complete the identified reasons in order to obtain a “peer-approved” list of reasons.*

*But the research questions also bear weaknesses: If these questions would have been formulated in a more concrete manner more valuable insights could have been revealed. Further, the authors made not full use of the strengths of the qualitative methods employed. As a consequence the results remain needlessly superficial: It seems highly likely that new insights can be revealed from the data if additional data analysis steps would be taken.*

*On the whole I recommend to accept the manuscript for publication after a major revision.”*

Thank you very much for your detailed comment. According to your advice, which is really helpful, we did a major revision and included a more detailed analysis. Unfortunately, we cannot change the research questions. We think that the type of our questions fits well with the aim of the study.

*2 Examples and evidence*

*2.1 Major issues*

*“There is one major issue which should be addressed by the authors primarily. Before I begin with the major issue I present two minor issues because all three of them are intertwined. Further minor issues follow in the next section.*

*I suggest changing the manuscript title from “factors of choice in dental care treatment” to “reasons for (not) choosing dental care treatment”. “Factors of choice” seems misleading since (a) it could be understood as “factors which should be chosen”; (b) it insinuates a quantitative approach instead of a qualitative one: In the manuscript the researchers do not analyse causal factors derived statistically but subjective reasons which people give for their actions.*

Thank you very much for the suggestion. We changed the manuscript title to “Reasons for (not) choosing dental treatments – A qualitative study based on patients’ perspective”.

*I suggest changing the first research question from “What factors influence patients in Germany in their decision on dental treatment?” to “What reasons do patients in Germany give for choosing or not choosing dental treatment?”. Mentioned themes found in transcripts are interesting per se but the elephant in the room is the question whether these themes represent reasons in favour of or against health care utilisation. I suggest changing the second research question from “How do patients assess cost coverage of dental services by German statutory health insurance?” to “What do patients think about cost coverage of dental services by German statutory health insurance?”. The term “assess” is ambiguous: It could mean “how do they think about cost coverage” (in the sense of “associations/ideas”) or it could mean “how do they evaluate cost coverage” (in the sense of “good/bad”). The reported results imply that the former interpretation seems adequate.*

We found your suggestion very helpful and changed the wording of research question no.1 “(1) What reasons do patients have for choosing or not choosing a dental treatment?” but kept the wording of research question no.2 since we think “assess” fits well. The wording in the manuscript regarding the research question no.1 was adjusted accordingly.

*One major issue arises from the data analysis. The manuscript text in the results section indicates that the focus group participants did not only name themes which revolve around dental health care utilisation but also whether the mentioned aspects represent reasons for choosing or not choosing dental treatment. It seems to suggest itself that this crucial dichotomy should have been coded and analysed as well: Firstly, I propose to use the additional codes “reason for choosing dental treatment” and “reason for NOT choosing dental treatment”. Secondly, it should be analysed which of the already identified reasons occur mainly in conjunction with the code “reason for choosing dental treatment” and with the code “reason for NOT choosing dental treatment” – and which reasons seem to be ambivalent because some people give these reasons for choosing dental treatment and others for NOT choosing dental treatment. It would be heuristically instructive if the authors would visualise the results like in figure 2 including the categories “group of themes mainly interpreted as reasons for choosing dental treatment” and “group of themes mainly interpreted as reasons for NOT choosing dental treatment” and a third category “group of ambivalent themes, sometimes interpreted in favour of and sometimes against dental treatment”.*

*This is an important issue: In the realm of quantitative research it would be like having large-scale survey data suited for multivariate inferential statistical analysis and analysing only descriptive frequency distributions and cross-tabulations.”*

We strongly support the idea to further categorize the identified findings into “reasons for choosing dental treatment” and “reasons for NOT choosing dental treatment” and therefore continued the data analysis. We rescreened the statements already given in the subcategories for each reason. We used the already existing codes and sorted the participants’ statements according to the reason for choosing and not choosing a dental treatment. If a statement could not be clearly assigned, it was defined as "unclear". In the following step, we added information on the interview questions to the statement. The “unclear” statements were screened again and mostly (re)sorted to “reasons for choosing a dental treatment” and “reasons for NOT choosing a dental treatment”. The screenings were performed independently by the researchers (SF, MB) for the statements of interview group 1 as a pilot. Intercoder agreement of r≥0.8 was considered as acceptable. Consensus was reached and SF continued sorting for interview groups 2 to 4. The number of statements was then counted and analyzed. We added an overview on statements distributions to the supporting information (S7 Table). However, we decided to use a reader-friendly table instead of listing the number of statements to the 53 reasons. According to the aim of qualitative research, we think this is a good way of presenting our additional results.

The methodological procedure and findings were added to the methods section and the results section and are further discussed in the discussion section of the manuscript. One finding is that many reasons can be classified as ambivalent, i.e., they are used for and against choosing a dental treatment.

*2.2 Minor issues*

*“In general, “factors of choice in dental treatment” should be replaced by “reasons for (not) choosing dental treatment”. Is the term “performance” the adequate term for the subcategories and codes which this category contains? “Health care service” seems to comprise the meanings better.*

*The detailed excursus on the coverage of dentures in Germany should be explicitly framed as additional information so readers have more context to understand the results (if that is its purpose). Otherwise this excursus does not seem relevant for the manuscript since neither the data generation process (focus groups) nor the data analysis process nor the results focus on dentures.*

*The methods should be explained in more detail, to make the way of the data more comprehensible, which steps were taken and so on. The authors should take heed of the “Standards for Reporting Qualitative Research: A Synthesis of Recommendations” by O’Brien et al. (2014). Especially, (a) they should reflect more on the sampling strategy: Why were participants recruited that way and what are the implications of this approach? (b) For the readers the main questions from the manual for the focus groups are necessary to gain a better grasp of the data generation process. (c) Relevant excerpts from the codebook or coding-scheme could be shown to retrace the data analysis process.*

*Data availability is limited due to participant privacy. This is a common issue with qualitative data but the authors offer access to anonymised interview transcripts on demand.*

*Finally, further proof-reading is necessary: There are still some orthographical and grammatical errors left.”*

Thank you for the helpful comments. We replaced the wording “factors of choice in dental treatment” by “reasons for (not) choosing dental treatment/s” (or “factor”/”factors” by “reason”/”reasons”) throughout the whole manuscript including figures, tables, the supporting information and the corresponding titles. We also replaced the word “performance” by “health care service” in the different sections. Further, we added the wording “Excursus:” to the heading of the paragraph giving additional information on coverage of dentures in Germany. Also, we added a short introduction to this paragraph to clearly outline that this section gives additional information (p.4 in the manuscript).

We now follow the criteria of the COREQ (COnsolidated criteria for REporting Qualitative research) checklist [published in “Consolidated criteria for reporting qualitative research (COREQ): a 32-item checklist for interviews and focus groups” by Tong et al. (2007)]. Accordingly, we partly extended the manuscript, e.g. methods section. We filled out in the COREQ checklist and provide it as supporting information (S1 File). In this respect, we added information about the interviewers/researchers and our data analysis process in more detail. Furthermore, we added information on our sampling strategy. Also, we added the interview guide to the supporting information (S1 Table). We used the recommended article of O’Brien et al. (2014) among other literature to report our research in the best possible way.

By signing the informed consent, interview participants confirmed that anonymized quotes based on the anonymized transcripts may be used for publication as part of the research project. In compliance with this requirement, we give single statements of the interview participants in the manuscript (Table 2, p.13; Fig 2) and the supporting information (S8, S9 and S10 Files, and S5 Table) for traceability and support of our study findings. We are not allowed to offer access to anonymized interview transcripts in another format, extend, or on demand. With the aim to give another opportunity to reproduce our data analysis process we provide

- a schematic presentation of the coding-tree, and the coding scheme including our codebook in the supporting information (S2 Table),
- the descriptive collection and analysis of “most”-important reasons (S7 File)
- the data base for calculating the intercoder-agreements of sorting statements (S8 and S9 File), and
- the data base for analysis of distribution of sorted statements on reasons (S10 File).

We have proof-read the manuscript again and corrected typographical and grammatical errors.
