# Supplementary material for: Reasons for (not) choosing dental treatments—A qualitative study based on patients’ perspective
Source: PLoS One. 2022 May 25;17(5):e0267656. doi: 10.1371/journal.pone.0267656 (PMC9132305; doi:10.1371/journal.pone.0267656)
Supplement: Attachment — Submitted filename: Manuscript Number PONE-D-20-35002.docx [file pone.0267656.s019.docx]

Manuscript Number PONE-D-20-35002

"Reasons for (not) choosing dental treatments – A qualitative study based on patients’ perspective"

The present manuscript highlights certain factors that influence patients in Germany in their decision on dental treatment and the way patients assess cost coverage of dental services by German statutory health insurance.

Focus group interviews - that were included in the study design - can be considered valuable tools in identifying issues that are more difficult for the community to become aware of. Such an approach allows the acquisition of "unfiltered" data directly from real life.

The authors reworked and analysed the data presented in the original manuscript and rewrote important parts of it, according to the reviewers’ suggestions.

The authors have described and clearly explained in the manuscript the limitations of this study, (e.g., regarding the selection of participants: “ […] certain behaviors concerning the utilization of dental treatments […] may differ between regions”). The authors have also mentioned that dental care of patient subgroups (e.g., children, patients with depression disease) should be part of future research.

Additionally, the authors replaced the wording “factors of choice in dental treatment” by “reasons for (not) choosing dental treatment/s” (or “factor”/”factors” by “reason”/”reasons”) throughout the whole manuscript including figures, tables, the supporting information and the corresponding titles. All these mentioned modifications are welcome.

Regarding Data Availability - the authors mentioned and explained that some restrictions will apply.

The manuscript is technically sound, and the data support the conclusions. The study design and the included analysis have been performed appropriately, the manuscript is presented in an intelligible fashion and written in standard English.
